# Design of an ultra-thin steerable probe for percutaneous interventions and preliminary evaluation in a gelatine phantom

**DOI:** 10.1371/journal.pone.0221165

**Published:** 2019-09-04

**Authors:** Marta Scali, Paulien A. H. Veldhoven, Paul W. J. Henselmans, Dimitra Dodou, Paul Breedveld

**Affiliations:** Department of Biomechanical Engineering, Faculty of Mechanical, Maritime and Materials Engineering, Delft University of Technology, Delft, The Netherlands; Vita Salute University of Milan, ITALY

## Abstract

Needles with diameter under 1 mm are used in various medical applications to limit the risk of complication and patient discomfort during the procedure. Next to a small diameter, needle steerability is an important property for reaching targets located deep inside the body accurately and precisely. In this paper, we present a 0.5-mm prototype probe which is able to steer in three dimensions (3D) without the need of axial rotation. The prototype consists of three Nitinol wires (each with a diameter of 0.125 mm) with a pre-curved tip. The wires are kept together by a stainless steel tube. Each wire is clamped to a block which translates along a leadscrew, the rotation of the latter being controlled by a wheel connected at the distal end of the leadscrew. The tip bends upon retraction of one or two wires. When pushed through a soft solid structure (e.g., a soft tissue or soft tissue phantom), the probe deflects due to off-axis forces acting on its tip by the surrounding structure. We tested the performance of the prototype into a 10% wt gelatine phantom, in terms of the predictability of the steering direction and the controllability of the final position after steering inside the substrate. The results showed that the probe steered in the direction of the retracted wire and that the final position varied from small deflections from the straight path when the wires were slightly retracted, to sharp curvatures for large wire retraction. The probe could be used in various applications, from cases where only a small correction of the path in one direction is needed to cases where the path to be followed includes obstacles and curves in multiple directions.

## Introduction

Medical needles are used in a variety of percutaneous procedures, including biopsy [1], drug delivery [2], and ablation [3]. Accurate and precise needle placement is required for the success of these procedures. During insertion, the needle can be misplaced due to tissue deformation and respiratory motion [4]. Often, in order to correct their trajectory, needles have to be withdrawn and re-inserted, which increases the procedure time and patient discomfort [5]. Additionally, poor control of the position and trajectory of a needle entails a risk of puncturing sensitive structures, such as blood vessels [6, 7].

### State-of-the-art steerable needles and probes

Over the last years, steerable needles and probes have been investigated in scientific works to enhance manoeuvrability through soft tissue [8–10]. Steerability helps the physician to correct misalignments and to reach the desired target while avoiding sensitive structures without the need of multiple insertions [11, 12].

A number of designs of steerable needles and probes have been proposed (see [9] for a review). A common steering method is by means of a needle tip with a pre-defined shape. Needles of this type typically consist of a cylindrical tube (body) and a tip with a bevel angle (i.e., bevel-tip needles) or a curve (i.e., pre-curved needles). Needles with multiple pre-curved cylindrical tubes combined in a telescopic way [13, 14] and needles with a tip that is both pre-curved and bevelled [15] have also been reported, and it has been shown that the latter achieves sharper steering curvatures than solely pre-curved or bevel-tip needles [16, 17]. Thanks to their simple design, it is possible to manufacture pre-defined shape needles with diameters smaller than 1 mm (see Table I in Scali et al. [18]). Konh et al. [19] tested bevel-tip needles with diameters of 0.38 mm, 0.51 mm, and 0.64 mm in gelatine phantoms and reported a maximum deflection of 36 mm achieved with the 0.38-mm needle at an insertion depth of 150 mm. Majewicz et al. [16] showed that a 0.58-mm pre-bent bevel-tip needle could bend inside goat liver with a small radius of curvature (34 mm), whereas a bevel-tip needle with the same diameter achieved a radius of curvature of around 667 mm, that is, a much flatter curve than the pre-bent bevel-tip needle.

A drawback of needles with a pre-defined shape is that the user does not have direct control over the needle curvature. Needles with a bevel tip rely on tissue interaction forces to steer. Steering in 3D is achieved with rotation of the needle body around its own axis to re-orient the bevel-tip towards the desired direction. In order to move straight, the needle has to be continuously rotated while being pushed forward. However, rotating a flexible long needle inside a soft organ generates torsional friction on the needle body, which might result in discrepancy between the orientation of the needle tip and the needle body [20]. This discrepancy can cause a loss of control over the needle path [21]. Additionally, it has been shown that when a bevel-tip needle is continuously rotated, it opens a spiral track in the soft organ, which increases the risk of tissue damage [22]. Pre-curved needles are usually consisting of a pre-curved stylet that is fed through a straight cannula. When the stylet is inside the cannula it is forced to assume a straight shape, once the stylet is pushed out of the cannula, it regains its pre-set curve. When the pre-curved stylet is inside the tissue, the user cannot directly correct its position; the only way to do this is to retract the stylet inside the cannula, rotate it, and reinsert it into the tissue. This method not only increases the time of the procedure, but could also damage the surrounding tissue due to the creation of multiple paths [20].

There are also needles that can steer by means of cable actuation. This mechanism allows on-demand steering at the tip in one plane [23, 24] or in two perpendicular planes [25, 26] and therefore changing the direction of the needle trajectory ‘on the spot’. Cable-actuated needles have a central backbone (i.e., the needle body), which is bent by pulling flexible elements connected to it (i.e., cables). The cables are generally placed at the maximum possible distance from the neutral bending line in the needle centre to create a large moment arm so that the needle can bend with a relatively small actuation force [27]. The smaller the diameter of the needle, the smaller the generated moment arms and thus the higher the actuation force needed to generate sharp curvatures, resulting in high internal forces in the needle.

Rutigliano et al. [28] showed that a 21 G (0.82 mm) 17-cm long cable-driven steerable needle (Morrison, AprioMed AB, Uppsala, Sweden) performed better than a 20 G (0.91 mm) 15-cm long straight needle with a bevel-tip (Chiba; Cook Medical, Bloomington, Ind) in terms of both procedure time and needle placement during fine needle aspiration. The steerable needle was composed of a rigid straight cannula and a steerable stylet that was actuated by a friction-based lever lock. The maximum tip deflection was 10 mm. The same needle was tested during a neural plexus blockade procedure to avoid traversing the kidney [12]. The idea was that the needle tip curves when the lever is turned and stays in position when the lever is released. However, the needle distal end did not remain curved after the stylet was removed, which is unwanted during accurate positioning of the needle. Adebar et al. [23] developed a flexural conical tip needle (tip Ø = 1.0 mm, body Ø = 0.8 mm) with a one-degree-of-freedom joint (Ø = 2.0 mm) actuated via a Nitinol pull wire. Ryu et al. [29] designed an active needle (Ø = 1.67 mm) where a superelastic Nitinol tube with laser machined slits could bend in one direction upon actuation of a shape memory alloy wire. Similarly, Gerboni et al. [24] presented an articulated needle (tip Ø = 1.35 mm, body Ø = 0.68 mm) with multiple flexural elements that allow the tip to bend in one direction when a tendon is pulled. These types of needles can change from a straight to a bent configuration once inside the tissue, but rotation of the needle body is needed to steer in 3D. The 2-mm diameter needle reported in Van de Berg et al. [25] used four actuation cables that run over a ball joint placed near the tip to deflect in two orthogonal planes, creating different steering trajectories once the needle is inserted into a substrate without the need of axial rotation. Summarizing, pre-defined shape needles can achieve small diameters due to their simple design which can be easily miniaturized (e.g., down to 0.4 mm [30]). However, pre-defined shape needles have limited steerability and usually require extra rotation of the needle body to steer in 3D. On the other hand, cable-actuated steerable needles allow for a high level of manoeuvrability, but they tend to have a more complex design than needles with a pre-defined shape. For this reason, the diameter of cable-actuated needles is usually larger than 1 mm.

### Aim

Reaching targets located deep inside the body while limiting tissue damage requires steerable needles that (1) have a small diameter (preferably under 1 mm) and (2) are able to 3D steering without the use of axial rotation. In this work, we aimed to combine the manoeuvrability of the cable-actuated needles with the easy-to-downscale design of the pre-defined shape needles and accordingly design a miniaturized probe with a diameter of 0.5 mm (25 G) suitable for omnidirectional steering in 3D without the use of axial rotation. First, the design process of the steerable probe is described, from the design requirements to the manufacturing of the probe prototype. Next, an experimental evaluation of the prototype in homogeneous tissue-mimicking phantoms is presented. A preliminary version of this work has been briefly reported in a conference abstract [31].

## Design process

### Design requirements

The probe should fulfil the following requirements:

1. The diameter should be under 1 mm. Typical diameters of a pre-defined needle found in literature are between 0.4 mm [30] and 0.9 mm [32] (see Table I in Scali et al. [18] for more examples).
2. The probe should be able to steer in 3D, with steering curvatures comparable to values presented in literature [18], where 34 mm is the smallest reported radius of curvature for a pre-curved needle with a diameter of 0.58 mm [16].
3. Current steerable needles with a diameter equal to or smaller than 1 mm are only able to steer in one direction, that is, one-degree of freedom (1-DOF), and axial rotation is thus needed to steer in different directions. The designed probe should be capable of omnidirectional steering in 3D without axial rotation of the probe, to avoid discrepancy between the orientation of the probe tip and the orientation of the probe body [20].

### Probe design

To allow for sharp steering curvatures, we used a set of straight elastic elements connected at the tip. If one element is being pulled at, while the rest of the elements are kept in place, the system bends around a bending plane that runs through the central axis of the stationary elements. In this way, the moment arm is enlarged compared to that in a standard cable-actuated needle, because the neutral line is shifted to the outer periphery of the needle.

To enlarge the moment arm even more, we pre-bent the elements outward at the tip. By doing this, the bending plane is shifted further sideways, so that the moment arm increases compared to straight elements, resulting in smaller internal forces and larger bending angles.

Adding an outward pre-bent at the tip, however, can locally increase the diameter of the needle. To keep the diameter equal throughout the length of the needle, we ensured that the bent did not exceed the wall thickness of the shaft cover nor the thickness of the part that connects the elements at the tip.

The required number of pre-curved elements is a trade-off between the desired steering accuracy, the bending radius of the steering curvature, and the total diameter of the probe. In order to steer in 3D without axial rotation, a minimum of three elements is required. To fulfil our requirements of a diameter smaller than 1 mm and a small bending radius, we therefore opted for three elements.

**Fig 1:**
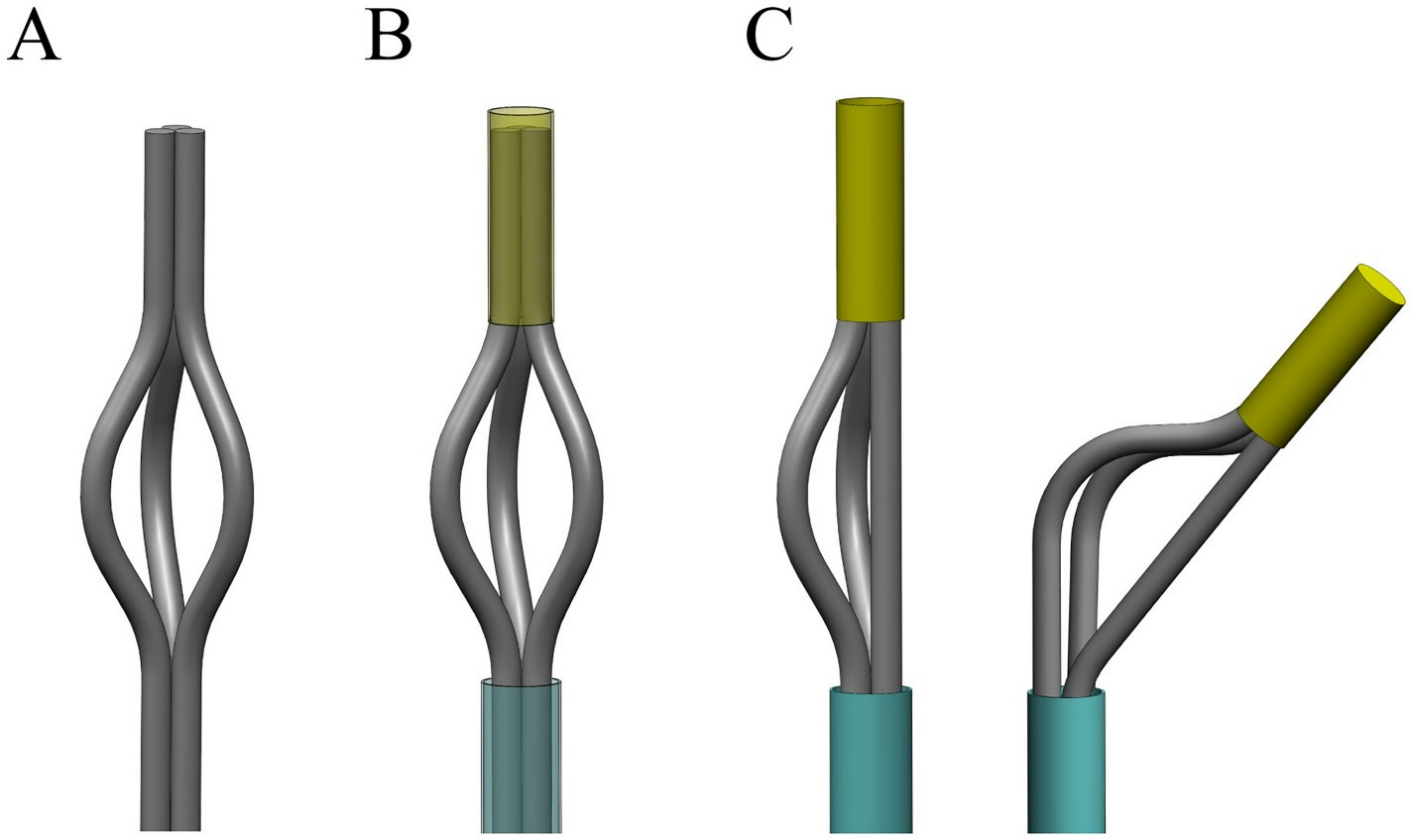
Schematic of the design of the probe tip. (A) Three elements with a pre-curve close to the tip. (B) The elements are connected at the tip and kept together along the body with a tubular sheath. (C) The element pulled is straightened and helps the other two pre-curved elements to bent, bringing the tip in a bent position.

Our final probe tip design consists of three flexible pre-curved elements connected together at the tip and covered with a sheath along their body (Fig 1A and 1B). Pulling one element straightens that element and gives freedom to the non-retracted elements to bend (Fig 1C). Additionally, the pulling element acts as a pulling cable, generating a force at the tip that helps the other two elements to bend. The bent elements make the probe steer upon insertion into a substrate. The more an element is pulled, the more the other two elements bend and the sharper the generated curve is.

### Probe prototype

**Fig 2:**
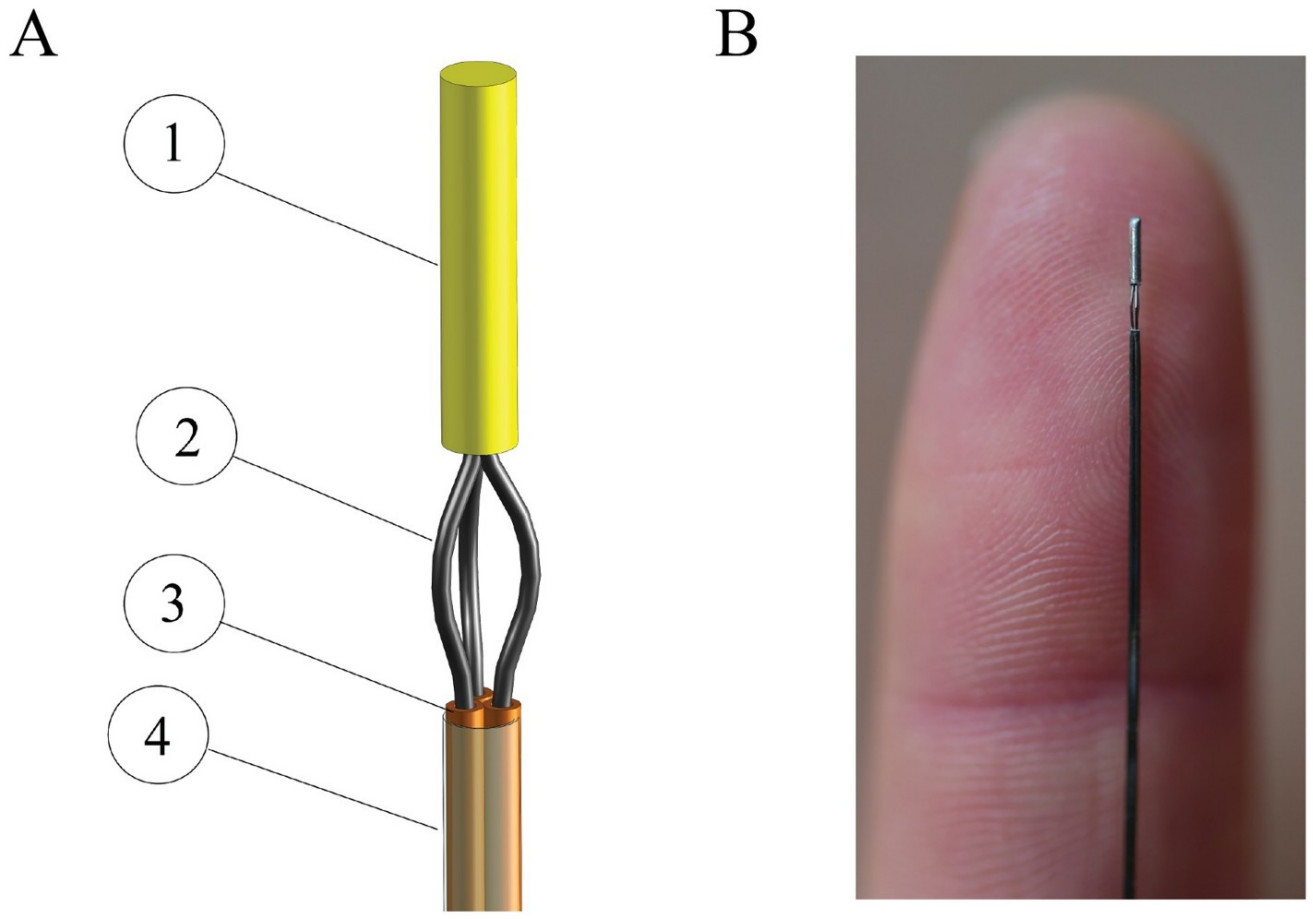
Probe tip. (A) 3D drawing of the probe tip showing the stainless steel tube (1), the Nitinol wires with the pre-curve (2), the Nitinol tubes (3), and the polyester shrinking tube (4). (B) A close-up of the probe tip in the straight configuration.

Our steerable probe (Fig 2) consists of three longitudinally aligned Nitinol wires (Ø = 0.125 mm). The wires are straight along their length and pre-curved at the tip. The curves at the tip are created by heating the wires up to 480 °C with a rework station (Tenma SMD rework station 220V), while being manually curved around a 0.3-mm diameter rod (for details on the choice of the needle tip design see S1 Appendix). A 3-mm long stainless steel tube (inner diameter (ID) = 0.3 mm, outer diameter (OD) = 0.5 mm) is glued at the tip, to keep the three wires together at their end points. The curved section of the wires below the tube has a length of 2 mm and a width of 0.5 mm. The other part of each individual wire is fed through an individual superelastic Nitinol tube (Johnson Matthey, ID = 0.15 mm, OD = 0.24 mm) up to the location of the pre-curve. The three Nitinol tubes (1) increase the bending stiffness of the probe shaft to facilitate insertion of the probe into a solid structure (e.g., soft tissue or soft tissue phantom), (2) guide the Nitinol wires without buckling from the probe base to the probe tip where the actual bending action takes place, similar to a Bowden cable, and (3) keep the wires in place over almost the entire needle length, preventing them from getting entangled. The tubes are fixed to the main body of the system, while the wires can slide freely through them. An ultrathin-walled polyester shrinking tube (Vention Medical, ID = 1.1 mm, wall thickness = 0.0076 mm) is put around the three Nitinol tubes, to keep them together without limiting their flexibility while only minimally increasing the diameter. The total diameter of the probe is 0.5 mm, the overall length of the steerable tip is 5 mm (i.e., the length of the stainless steel tube plus the length of the curves), and the total length of the probe is 180 mm.

**Fig 3:**
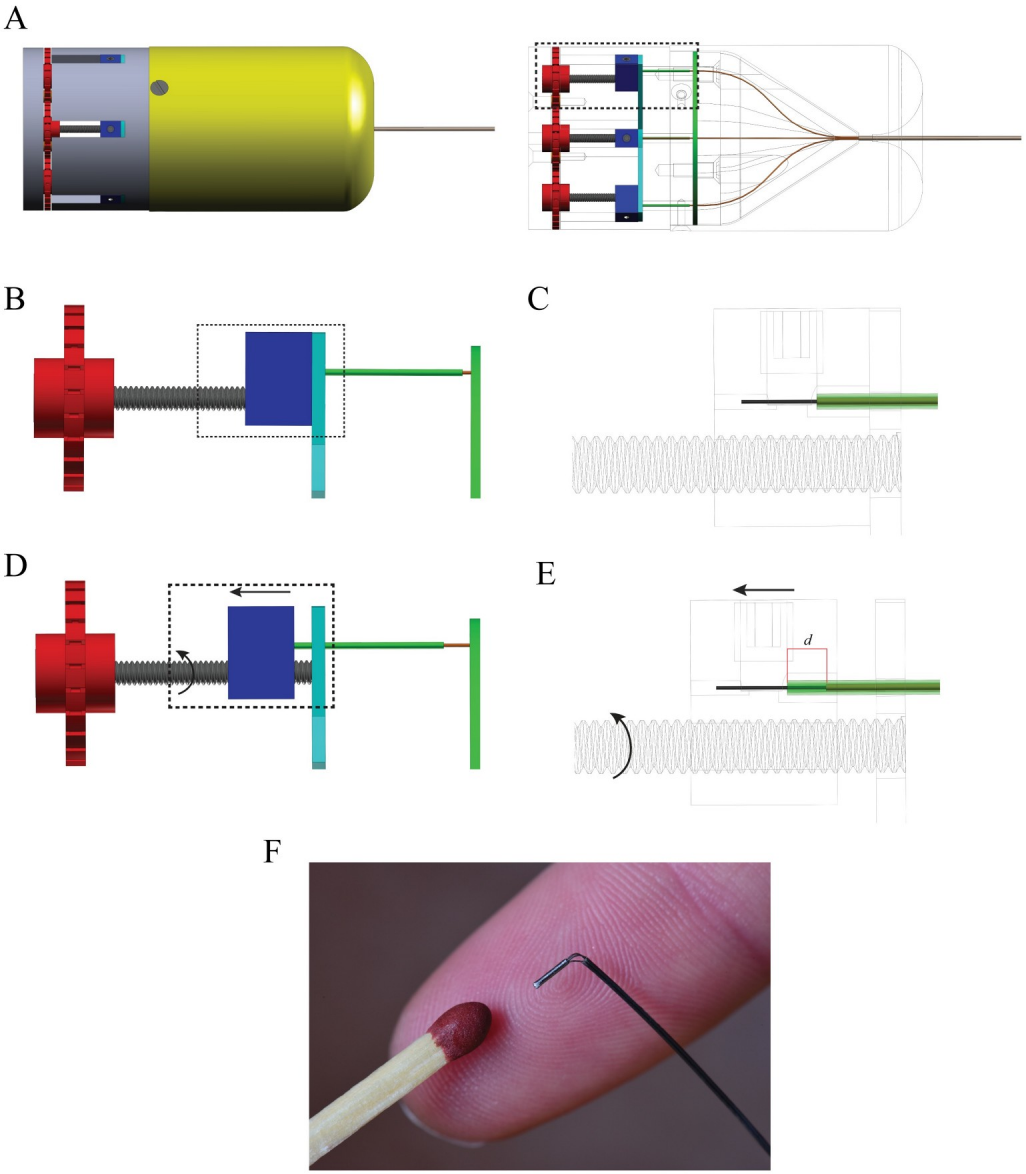
Actuation mechanism of the probe prototype. (A) 3D drawing of the actuation unit (left) and the leadscrew mechanisms with the wires surrounded by the tubes visible inside the main body (right). (B) Close-up of one leadscrew mechanism at the initial position. (C) Close-up of the wire clamped inside the block surrounded by the Nitinol tube (orange) and the rigid tube (green), which is glued inside the block. (D) Close-up of one leadscrew mechanism after the wheel has been turned and the block with the wires clamped inside translated. (E) Close-up showing the position of the wire, Nitinol tube, and rigid tube after the block translation. When the block translates, the wire is retracted leaving behind the Nitinol tube that is glued to the ring-shaped object (green). The rigid tube protects the length of the wire *d* that is not supported by the Nitinol tube. (F) Photograph of the tip bent after retraction of one wire. Dashed rectangular windows highlight the areas of interest. Arrows show the directions of motion of the parts.

The retraction of each wire is controlled by a manual leadscrew mechanism (Fig 3). Each wire is clamped to a block which can translate along a leadscrew, the rotation of which is controlled by a steering wheel connected at the distal end of the leadscrew. An M2x20 screw with a pitch of 0.4 mm is used, meaning that one full rotation of the wheel leads to a 0.4 mm translation of the block and the connected wire. There are large and small grooves on the wheels, serving as a visual indication corresponding to a 1/4 rotation (0.1 mm translation of the block) and a 1/24 rotation (0.017 mm translation of the block), respectively. When the block translates, a part of the wire is exposed and become prone to buckling. A rigid tube glued to the block through which the Nitinol wire with its surrounding Nitinol tube is fed prevents buckling.

**Fig 4:**
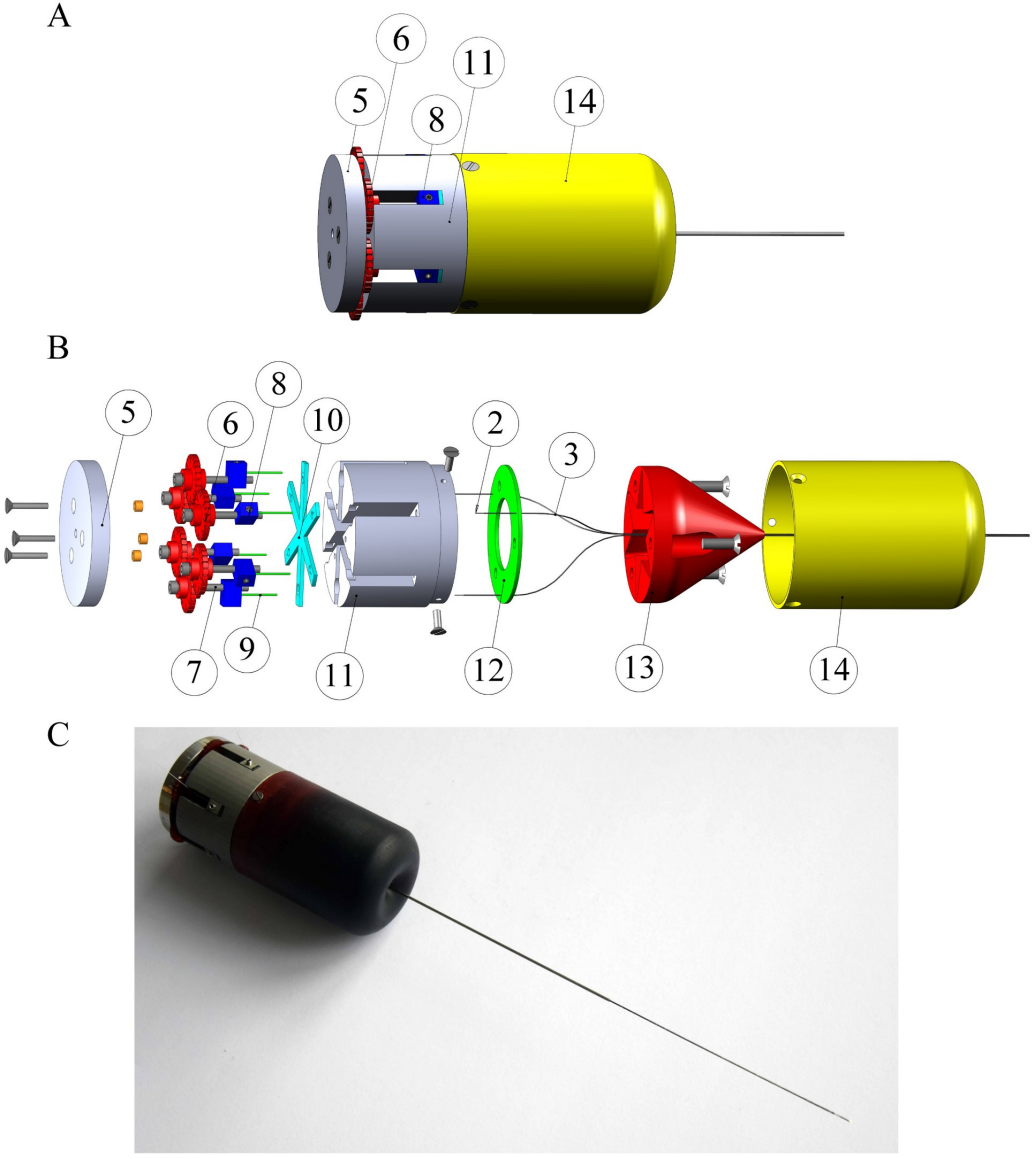
Prototype of the ultra-thin probe. (A) 3D drawing of the actuation unit showing the back plate (5), wheels (6), block (8), main body of the unit (11), and protection cap (14). (B) Exploded view of the actuation unit showing the back plate (5), wheel (6), lead screw M2x20 (7), block (8), rigid tube (9), star-shape plate (10), main body (11), Nitinol wire (2), Nitinol tube (3), ring-shaped object (12), conical guidance cap (13) and protection cap (14). (C) Photograph of the probe prototype.

The main body of the actuation unit has a cylindrical shape with rectangular slots to accommodate the blocks and prevent them from rotating (Fig 4). A star-shape plate is positioned between the blocks and the internal wall of the slot to guide the leadscrew. A guidance cap with inner grooves is used to guide the Nitinol tubes from the back to the front of the actuation unit, from where they are kept together by the shrinking tube for the whole length of the probe body. The Nitinol tubes are glued to a flat ring-shaped object positioned at the top of the main body and at the top of a guidance cap to prevent their motion. The actuation unit is covered with a protection cap with rounded edges to avoid damaging the probe at the point of entry into the actuation unit.

The body of the actuation unit is 3D printed in stainless steel (type 316L) using laser metal fusion (Sisma, type Mysint100). The wheels and the cap are 3D printed from polymeric material (acrylate, R05 red). The actuation unit is designed to accommodate up to six Nitinol wires.

## Experimental evaluation

We performed two experiments to evaluate the performance of the probe, assessing the predictability of the steering direction and the controllability of the final position after steering, respectively. The experimental setup was the same for both experiments.

### Gelatine phantom

The probe was tested in 10% wt gelatine phantoms. These phantoms were prepared by mixing hot water and gelatine powder (Dr. Oetker Professional, The Netherlands) and casting the mixture into transparent boxes (12 cm x 8 cm x 12 cm). The liquid gelatine was left at 4°C overnight to solidify.

The stiffness of 10% wt gelatine phantom was characterized using a rheometer (AR-G2 Rheometer, TA Instruments Ltd) with a parallel-disc geometry (diameter of 25 mm). First, the response of the gelatine to a strain change (range 0.01–10%) at a constant velocity of 10 rad/s was measured, to find the linear viscoelastic region of the material. After that, the response of the gelatine to a frequency change between 100Hz and 0.1 Hz at 0.5% strain (value within the linear viscoelastic region) was measured.

From these tests, we obtained values for the storage modulus (G′) and the loss modulus (G″). The moduli were averaged across frequencies to calculate the dynamic shear modulus (G* = G′ + G″). The absolute value of G* was calculated as $\text{G}=\sqrt{{\text{G}}^{\prime 2}+{\text{G}}^{\prime \prime 2}}$. The Young’s modulus E was estimated by the shear modulus G using the equation E = 2G(l + ν), where ν is the Poisson’s ratio (E = 3G, for ν = 0.5). Based on these data, we estimated a Young’s modulus of 17 kPa for 10% wt gelatine phantom. Usually, the Young’s modulus of the soft tissues is characterize by a range of values [33, 34], which might vary depending on the measurement method [35, 36]. The Young’s modulus of the gelatine in our study approximates skeletal muscle tissue (12–32 kPa) [37], healthy liver (10–20 kPa) [38], or breast glandular tissue (7.5–66 kPa) [39].

### Experimental setup

**Fig 5:**
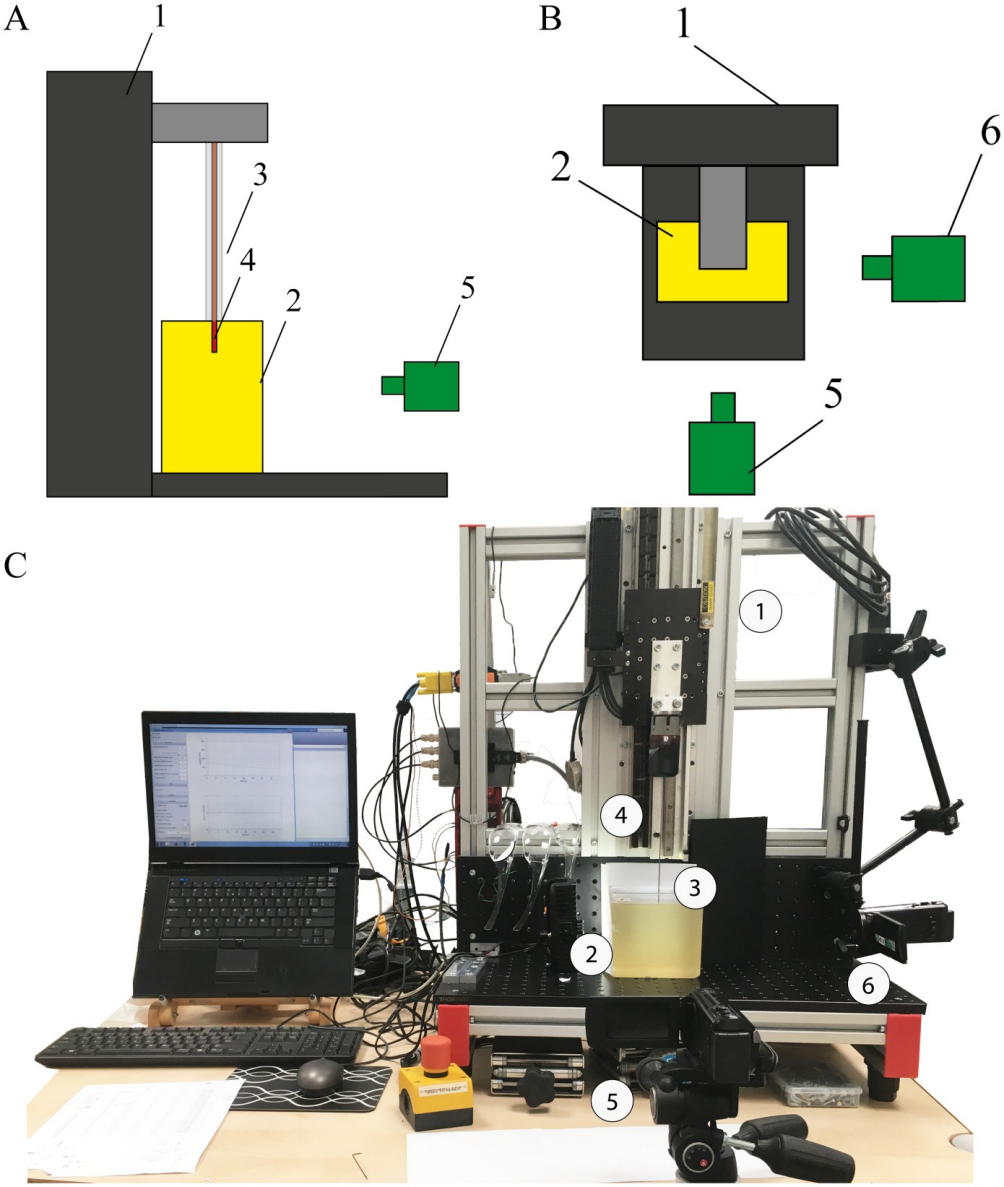
Experimental setup. (A) Schematic illustration of the setup in side view, with the linear stage (1), the gelatine box (2), the concentric tubes for the insertion of the probe (3), the probe itself (4), and the front view camera (5). (B) Schematic illustration of the setup in top view, with two cameras, the front view camera (5) and the side view camera (6). (C) Photograph of the experimental setup.

A linear stage (Aerotech ACT115, model MTC300) was used to insert the probe inside a block of gelatine with a constant speed of 2 mm/s. The probe was mounted on the linear stage by means of an aluminium block. To avoid buckling of the probe outside the gelatine during insertion, two concentric tubes were placed over the probe. The outer tube (OD = 2 mm, ID = 1.1 mm, length = 50 mm) and the inner tube (OD = 1 mm, ID = 0.6 mm, length = 65 mm) were both made of stainless steel. When the probe is pushed inside the gelatine, the tubes slide into one another, restricting the lateral motion of the probe. Two video cameras (Panasonic HC-V250) were used to acquire a front and a side view image during insertion (Fig 5).

### Actuation modes

**Fig 6:**
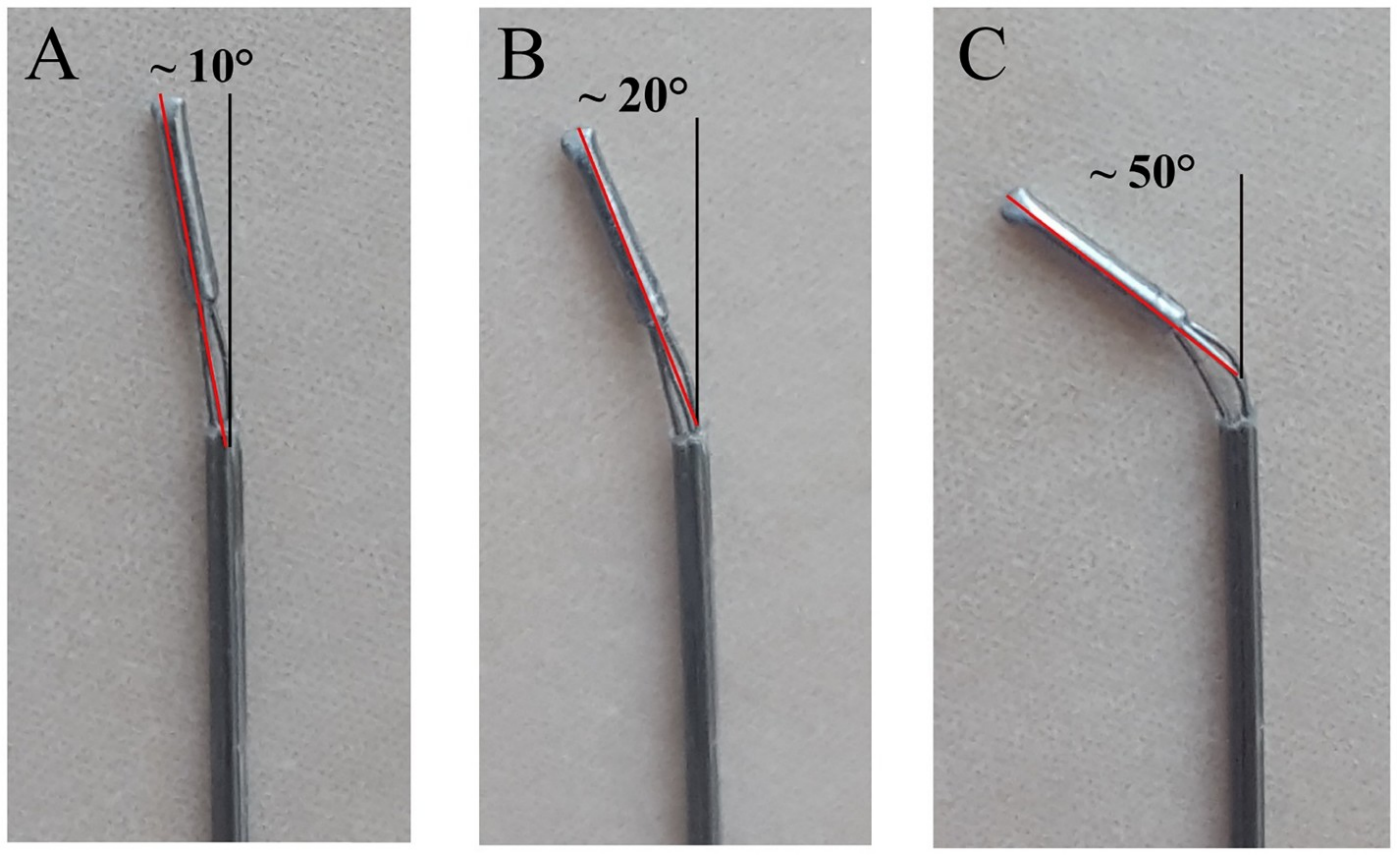
Position of the tip after the three level of retraction. (A) L1, (B) L2, and (C) L3 retraction (see text for the definitions of L1, L2, and L3).

The tip can be bent by pulling at one or more wires. In order to bent the tip, the operator rotates one or more of the wheels described in the previous section, which leads to the translation of the block in which the wire is clamped, and via that translation to the bending of the tip. The amount of pulling depends on the translation of the block, which is controlled by the number of turns of the wheel. In our experiments we distinguished three levels of pulling (Fig 6):

- Level 1 (L1): one turn of the wheel, which corresponds to a 0.4-mm block translation and 10° bending of the tip.
- Level 2 (L2): two turns of the wheel, which corresponds to a 0.8-mm block translation and 20° bending of the tip.
- Level 3 (L3): three turns of the wheel, which corresponds to a 1.2-mm block translation and 50° bending of the tip.

Note that these levels of pulling have been measured in free space. Once the needle is inserted into a substrate, the degree of tip bending might be smaller than these values, due to resistance forces by the tissue [29].

### Experiment 1: Steering direction

#### Experimental design and procedure description

In Experiment 1, the predictability of the steering direction was assessed as a function of which wire was retracted (W1, W2, or W3) and the number of wires that were retracted simultaneously (one or two). In order to measure the steering direction, we used a polar coordinate system, in which each point on a plane is determined by the distance from a reference point and the angle from a reference direction. The wires are positioned 120° from each other, therefore when one or two wires are retracted, the theoretical difference between the two resulting steering directions of the probe is 120°. The difference between the two resulting steering directions when one wire is retracted and when two wires are retracted is therefore 60°.

**Fig 7:**
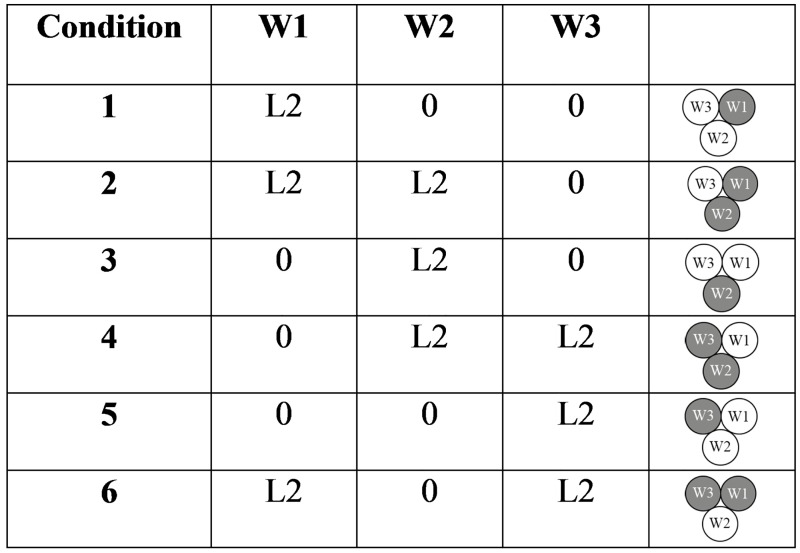
Conditions for Experiment 1. W1, W2, and W3 indicate which wire was retracted, and L2 is the chosen level of retraction in the experiment (0.8 mm).

The level of retraction was set at 0.8 mm (L2) for all conditions, because the steering direction is not expected to depend on the level of retraction. A total of six conditions were tested (Fig 7). Each condition was tested five times.

Before each test, the probe was inserted for about 10 mm inside the gelatine phantom using the linear stage and photographed with the front and side cameras. The initial insertion was done to reduce the effects of cutting and friction forces acting on the tip when punching the substrate. Next, the wheels of the actuation unit were turned to obtain the tip configuration corresponding to the desired condition. Once the tip was set, the linear stage pushed the probe inside the gelatine for 65 mm. At the end of each measurement, a photograph from both sides was taken. After that, the probe was retracted using the linear stage. All measurements were performed over the course of one day.

#### Data analysis

**Fig 8:**
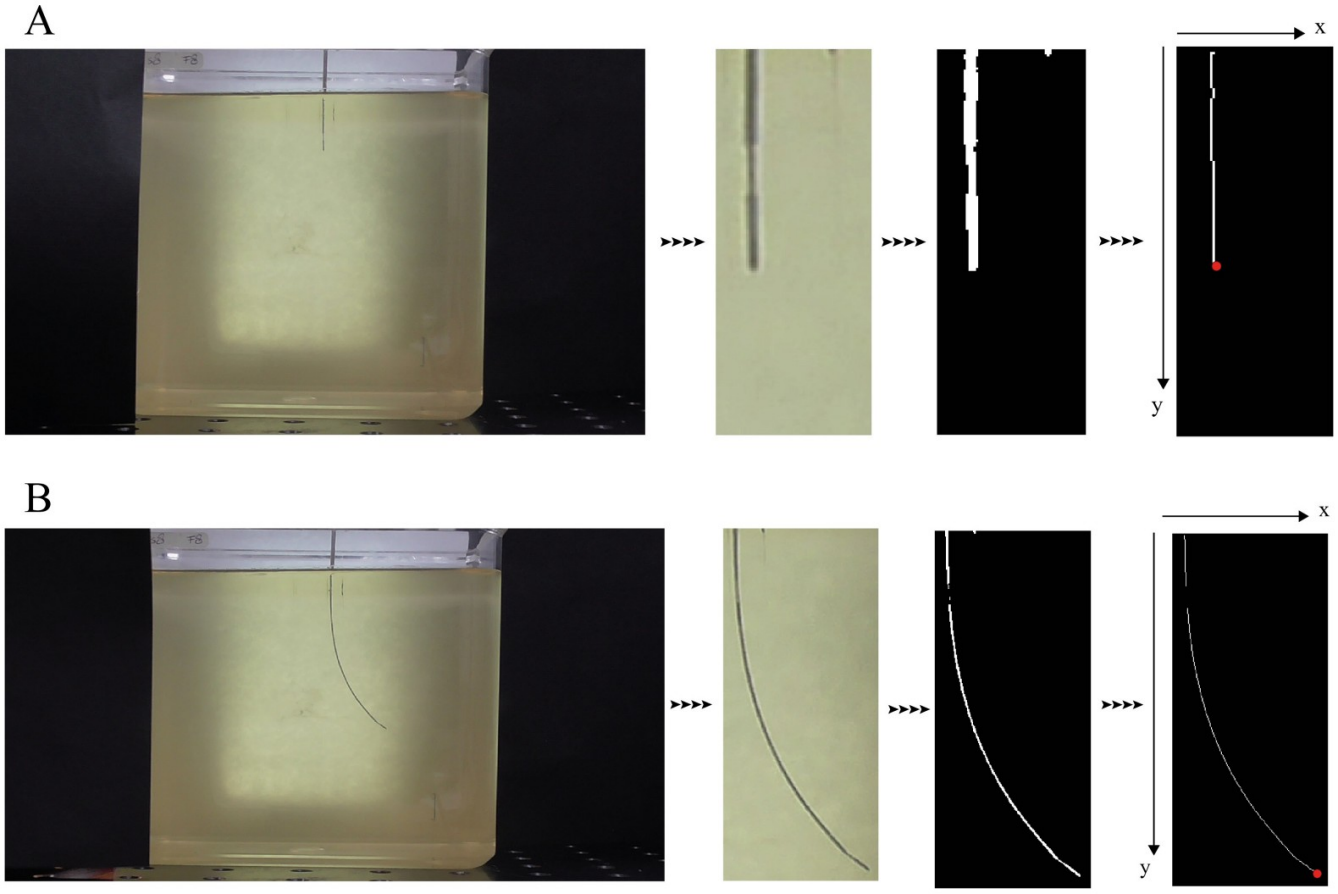
Image processing of the photographs. (A) Initial front view photograph. (B) Final front view photograph. In this example, we used the images for Condition 1, Measurement 3. First, the original photograph was read in MATLAB and cropped around the area of interest. After that, the photograph was converted to a binary image, and the skeleton of the image and the coordinates (x, y) of the last point of the probe were retrieved.

The photographs of the initial and final position of both front and side view were imported to MATLAB (R2016b). After cropping the photographs around the area of interest, we increased the contrast and converted them to binary images. Next, we extracted the skeleton of the binary images, from which we took the coordinates (x, y) of the last point of the probe (i.e., the probe tip) (Fig 8). After that, we calculated the difference between the x- and y-coordinates of the last point in the final image and in the initial image, for both front and side view as:

$$\begin{array}{ll} \Delta {\text{x}}_{\text{front}}={\text{x}}_{\text{front}}-{\text{x}}_{\text{INfront}} & \Delta {\text{x}}_{\text{side}}={\text{x}}_{\text{side}}-{\text{x}}_{\text{INside}} \end{array}$$

$$\begin{array}{ll} \Delta {\text{y}}_{\text{front}}={\text{y}}_{\text{front}}-{\text{y}}_{\text{INfront}} & \Delta {\text{y}}_{\text{side}}={\text{y}}_{\text{side}}-{\text{y}}_{\text{INside}} \end{array}$$

**Fig 9:**
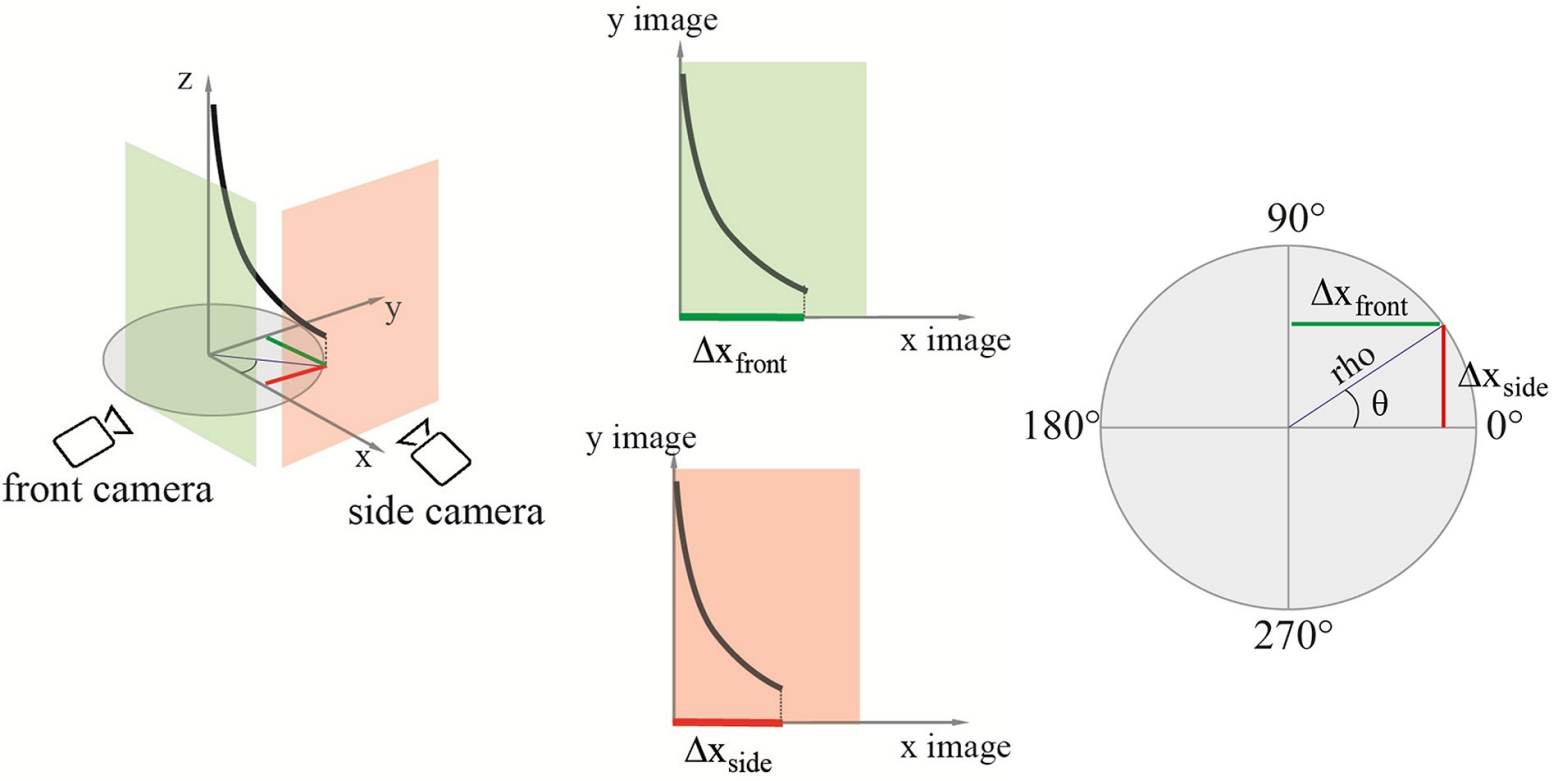
Schematic representation of the probe on the 3D plane. In red, the y-coordinate of the final point of the probe (Δx_side_). In green, the x-coordinate of the final point of the probe (Δx_front_). The angle θ is equal to arctan(Δx_side_[mm], Δx_front_[mm]), and the distance rho is $\sqrt{\left(\Delta {\text{x}}_{\text{front}}{\left[\text{mm}\right]}^{2}+\Delta {\text{x}}_{\text{side}}{\left[\text{mm}\right]}^{2}\right)}$.

Where (x_front_, y_front_) and (x_side_, y_side_) are the coordinates of the last point of the probe in the final image of the front view and side view, respectively, and (x_INfront_, y_INfront_) and (x_INside_, y_INside_) are the coordinates of the last point of the probe in the initial image of the front view and side view, respectively (Fig 9). The differences along the y-axis (Δy_front_ and Δy_side_) represent the projection of the final point reached by the probe along the axis of insertion in front and side view, and the differences along the x-axis (Δx_front_ and Δx_side_) represent the deflection in front and side view. To calculate the actual difference in mm we used a scaling factor (SF). The scaling factor for the front view (SF_front_) was estimated by using the length dimension of the gelatine box (l x w x h, 120 x 80 x 120 mm), knowing that the SF is equal to the box length in [mm] divided by the box length in pixels. The scaling factor of the side view (SF_side_) was estimated knowing that the depth of the box in front and side view should be the same.

$${\text{SF}}_{\text{side}}=\frac{\left(\Delta {\text{y}}_{\text{front}}\cdot {\text{SF}}_{\text{front}}\right)}{\Delta {\text{y}}_{\text{side}}}$$

From the coordinates of the probe tip we can calculate the angle θ, which indicates the direction of steering of the probe, and the distance rho, which indicates the radius of curvature of the circular path described by the probe (Fig 9). The value of rho can be used as a measure of the deviation of the probe from the straight path. We calculated the two parameters, θ and rho, as:

$$\theta =\text{arctan}(\Delta {\text{x}}_{\text{side}}[\text{mm}],\Delta {\text{x}}_{\text{front}}[\text{mm}])$$

$$\text{rho}=\sqrt{\left(\Delta {\text{x}}_{\text{front}}{\left[\text{mm}\right]}^{2}+\Delta {\text{x}}_{\text{side}}{\left[\text{mm}\right]}^{2}\right)}$$

### Experiment 2: Final position after steering

#### Experimental design and procedure description

**Fig 10:**
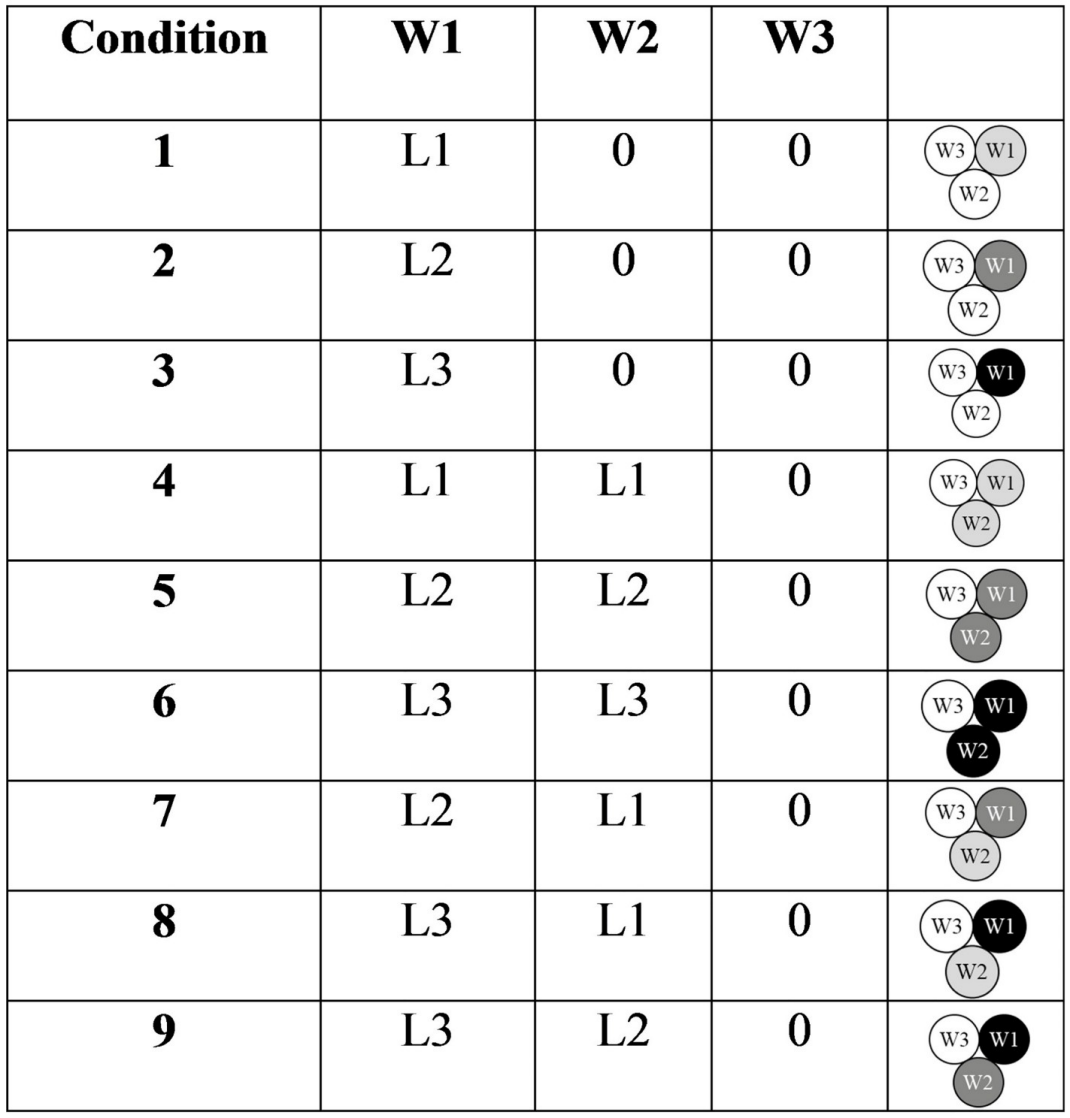
Conditions for Experiment 2. W1, W2, and W3 indicate which wire was retracted, and L1, L2, and L3 indicate the level of retraction (0.4 mm, 0.8 mm, and 1.2 mm). The three circles represent the three wires. Light grey, medium grey, and black indicate retraction by 0.4 (L1), 0.8 (L2), and 1.2 mm (L3), respectively.

In Experiment 2, the final position after steering was assessed as a function of the number of retracted wires (one or two), and the level of retraction (L1 = 0.4 mm, L2 = 0.8 mm, or L3 = 1.2 mm, corresponding to one, two, or three turns of the wheel, respectively). For one-wire retraction, W1 was retracted, and for two-wire retraction, W1 and W2 were retracted. The combinations used for the experiments are shown in Fig 10. The experiment was performed following the same procedure of Experiment 1.

#### Data analysis

The photographs were processed in MATLAB (R2016b) following the same procedure as described for Experiment 1. From the coordinates of the end point of the probe tip, we calculated the difference between the position of the end point in the initial image and in the final image. These values were used to calculate the angle θ and the distance rho. The angle θ indicates the direction of steering of the probe, and rho indicates the deflection of the probe from the straight path as described earlier. The final position after steering was assessed as a function of the amount of deflection from the straight path and the projection of the final point reached by the probe along the axis of insertion, called henceforth insertion depth. We used the ratio between deflection and insertion depth to evaluate the steerability of the prototype:

$$\text{ratio}=\frac{\text{deflection}}{\text{insertion}\;\text{depth}}$$

A small curvature was defined by a ratio < 1, meaning that the probe deflection was smaller than the insertion depth. A sharp curvature was defined by a ratio > 1, meaning that the probe deflection was larger than the insertion depth.

## Results

### Experiment 1

**Fig 11:**
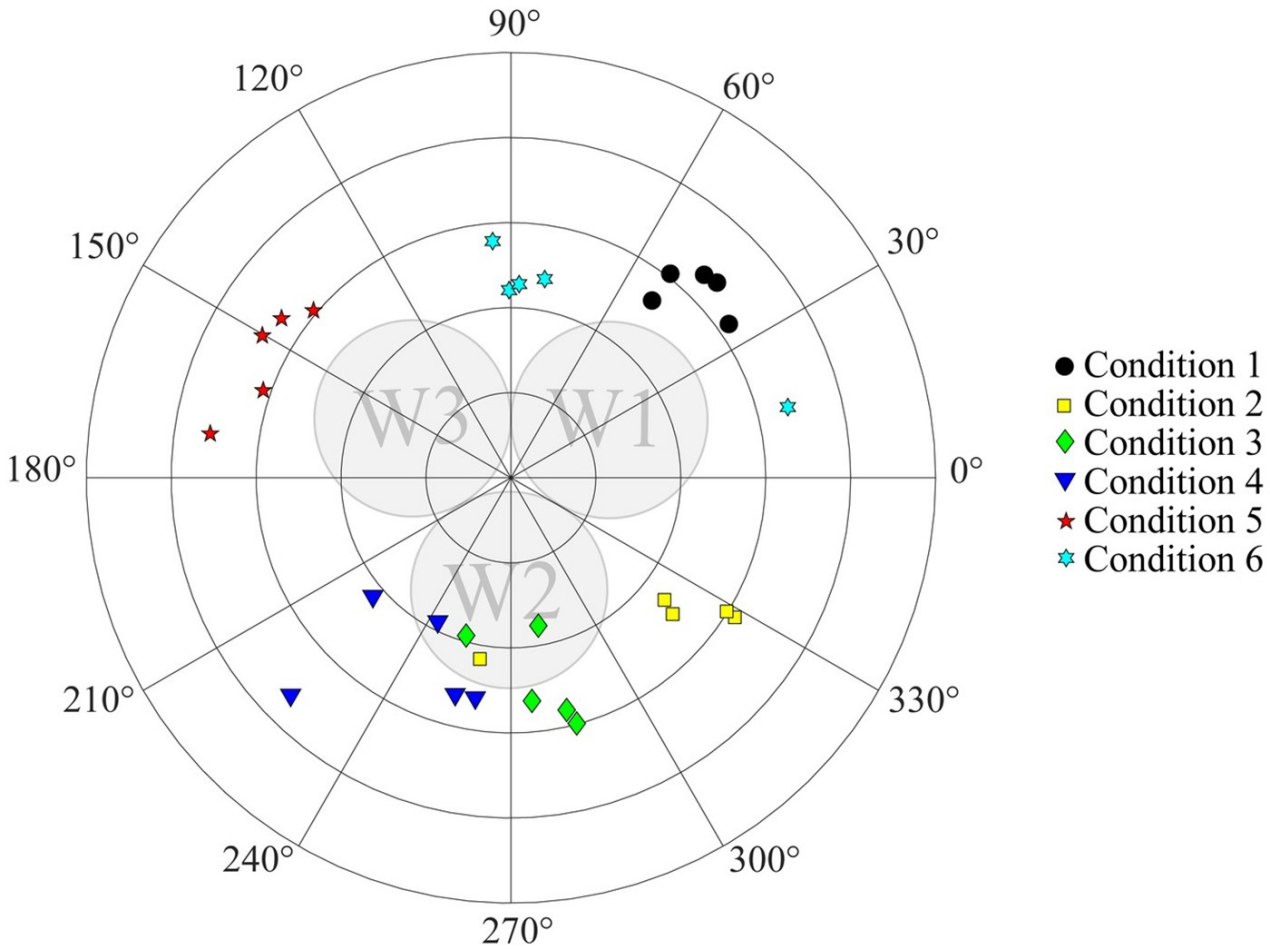
Polar plot showing the angle of the steering direction of the probe measurements.

Fig 11 shows the angles achieved for all (6x5) measurements. In Table 1, the median and interquartile range of the steering direction angles for each condition are presented. Table 2 shows the differences between the median values of the steering direction among the conditions. It can be seen that Conditions 1 and 4 had the lowest and the highest variability, respectively. The conditions in which two wires were retracted exhibited a larger variability than the conditions in which only one wire was retracted.

**Table 1:** Median and interquartile range of the steering direction angle for each condition.

Condition	Median	Interquartile range
**1**	46.2°	10.2°
**2**	321.6°	23.1°
**3**	280.5°	13.8°
**4**	243.0°	33.3°
**5**	150.3°	19.5°
**6**	87.6°	27.7°

**Table 2:** Differences between the medians of the steering direction angle among conditions.

**Conditions**	**1**	**2**	**3**	**4**	**5**
**2**	84.7°				
**3**	125.7°	41.0°			
**4**	163.2°	78.6°	37.5°		
**5**	104.0°	171.3°	130.2°	92.7°	
**6**	41.4°	126.0°	167.1°	155.4°	62.7°

### Experiment 2

In Condition 6, one out of the five measurements was excluded because the probe moved upwards during bending, meaning that the start and the final position could not be properly compared.

**Fig 12:**
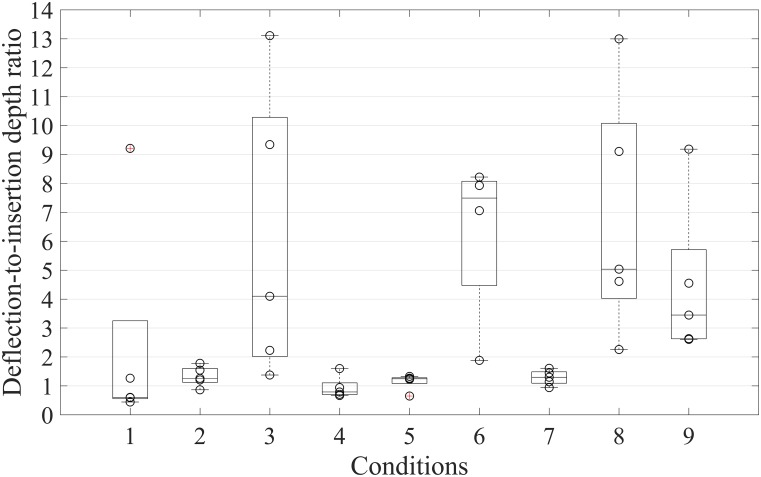
Deflection-to-insertion depth ratio for each condition. For Condition 6 only four out of five measurements are displayed.

The ratio between deflection and insertion depth was below 1 for Conditions 1 and 4, and above 1 for the remainder of the conditions (Table 3 and Fig 12). For one-wire retraction with L1 (Condition 1), the ratio was under 1 (0.60) and increased with an increasing level of retraction. The same holds for two-wire retraction with the same level of retraction (L1, L2, or L3). The ratio was 0.79 for W1-L1 and W2-L1 (Condition 4), 1.24 for W1-L2 and W2-L2 (Condition 5), and 7.49 for W1-L3 and W2-L2 (Condition 6). Condition 6 had the highest ratio value (7.49), followed by Condition 8 (5.03), and Condition 3 (4.09). Conditions 3 and 8 had the highest variance.

**Table 3:** Median and interquartile range (IQR) of the deflection, insertion depth and ratio deflection-to—Insertion depth for each condition.

Condition	Deflection [mm] Median (IQR)	Insertion depth [mm] Median (IQR)	Ratio Median (IQR)
**1**	33.25 (14.71)	51.95 (29.54)	0.60 (2.69)
**2**	47.36 (5.35)	36.21 (9.87)	1.26 (0.48)
**3**	49.45 (8.17)	13.52 (21.03)	4.10 (8.27)
**4**	39.41 (6.54)	48.70 (11.58)	0.79 (0.41)
**5**	43.70 (4.41)	35.14 (6.23)	1.24 (0.20)
**6**	56.43 (16.10)*	8.04 (10.89)*	7.49 (3.60)*
**7**	45.49 (6.41)	35.10 (7.31)	1.30 (0.39)
**8**	50.27 (29.99)	9.77 (4.96)	5.03 (6.05)
**9**	53.20 (6.70)	15.66 (8.35)	3.45 (3.08)

*number of measurements n = 4

## Discussion

In this study, we presented a new design of a steerable probe that is able to steer in 3D without the need of axial rotation. With a diameter of 0.5 mm (25 G), this is the smallest reported probe that steers in 3D without the need of axial rotation [18]. Combining the simple design of a pre-curved needle [40] with the steering capability of a cable-actuated steerable needle [25] allowed us to miniaturize the needle without losing the omnidirectionality of the steering. Our probe does not have any additional means of actuation; the Nitinol wires, which the probe consists of, act both as pre-curved elements and as pulling cables.

Once the probe with the bent tip is inserted into the gelatine phantom, the probe follows a curved trajectory due to off-axis reaction forces generated at the tip. The probe is able to steer omnidirectionally following the direction of the retracted wire(s), and the sharpness of the curvature increases with the level of wire retraction.

### Probe performance

Our aim was to evaluate the performance of the prototype in terms of predictability and controllability of steering. In order to know the path followed by the probe, direct vision of the probe inside the phantom was needed. Options to do this include (electromagnetic) tracking and direct visualization from outside the phantom. Since the probe was not designed to contain sensors at the tip or in the body, direct visualisation was chosen, for which a transparent substrate (i.e., gelatine) with mechanical properties similar to soft tissue was used. In literature, gelatine, polyvinyl alcohol, and agar are commonly used to mimic tissue [41, 42], because of their versatility in terms of shape and stiffness modification. Doing the tests in such controlled environment allowed us to get data on the performance of the prototype and set the requirements for future improvements of the prototype.

In Experiment 1, we assessed the predictability of the steering direction as a function of which wire was retracted. The probe performed more accurately (i.e., exhibited less variation) for one- than for two-wire retraction. One possible explanation is that, in order to achieve the direction wanted with two-wire retraction, the wires should be retracted by the same amount. The actuation was done manually and there was no locking of the system in position once the wires were pulled. This might have caused mismatch between the input (wheel rotation) and the output (tip bending). If one element is retracted more than the other one, the steering direction is not exactly in the middle of the two retracted wires but rather shifted towards the direction of the wire that is more retracted. Additionally, when one wire is retracted, the bending force generated is in line with the direction of the desired bending motion, whereas when two wires are retracted, the bending forces are not in line with the direction of motion making the system unstable.

In Experiment 2, we assessed the final position after steering as a function of the deflection and the insertion depth. The deflection-to-insertion depth ratio increases with the level of retraction of the wire. This behaviour can be seen in both one-wire retraction and two-wire retraction. The more a wire is retracted, the sharper the bend and the higher the deflection-to-insertion depth ratio. This result is in line with observations in the literature. Specifically, Wedlick et al. [43] analysed the curvature of a series of pre-curved needle with various arc lengths. These authors showed that a needle with a large arc length follows curves with a smaller radius of curvature than a needle with a small arc length. Sitzman et al. [44] compared the deflection of needles with diameters of 0.64 mm (22G), 0.47 mm (25G), 0.36 mm (27G), and 0.28 mm (29 G) and with a 5° versus a 10° bend. At 60 mm depth, the deflection with the 10° bend was greater than the deflection of the needle with 5° bend, and this deflection increased for smaller needle diameters. Increasing the tip angle is not always related to an increase of the needle curvature. Adebar et al. [23] analysed the influence of the tip angle on curvature of a bent-tip needle. They used three needles (diameter = 0.8 mm) with a tip angle of 30°, 45°, and 60° and tip length of 12 mm. The curvature of the needle increased when increasing the tip angle from 30° to 45°, whereas there was no change in the curvature when increasing the tip angle further, from 45° and 60°. Additionally, our results showed that the variability of the ratio between deflection and insertion depth among conditions is relatively high compared to state-of-the-art needle designs. However, in literature, 4-mm pre-bent tip length also showed a large variability in deflection [16]. A way to reduce this variability could be to increase the pre-bent tip length [23].

Condition 6 exhibited the highest deflection-to-insertion depth ratio (7.49), followed by Condition 8 (5.03) and Condition 3 (4.09). These are the conditions in which wire 1 was retracted with the highest level of retraction (L3 = 1.2 mm), which means that the tip was highly bent (~ 50°), resulting in high curvature once the probe was pushed through the gelatine.

**Fig 13:**
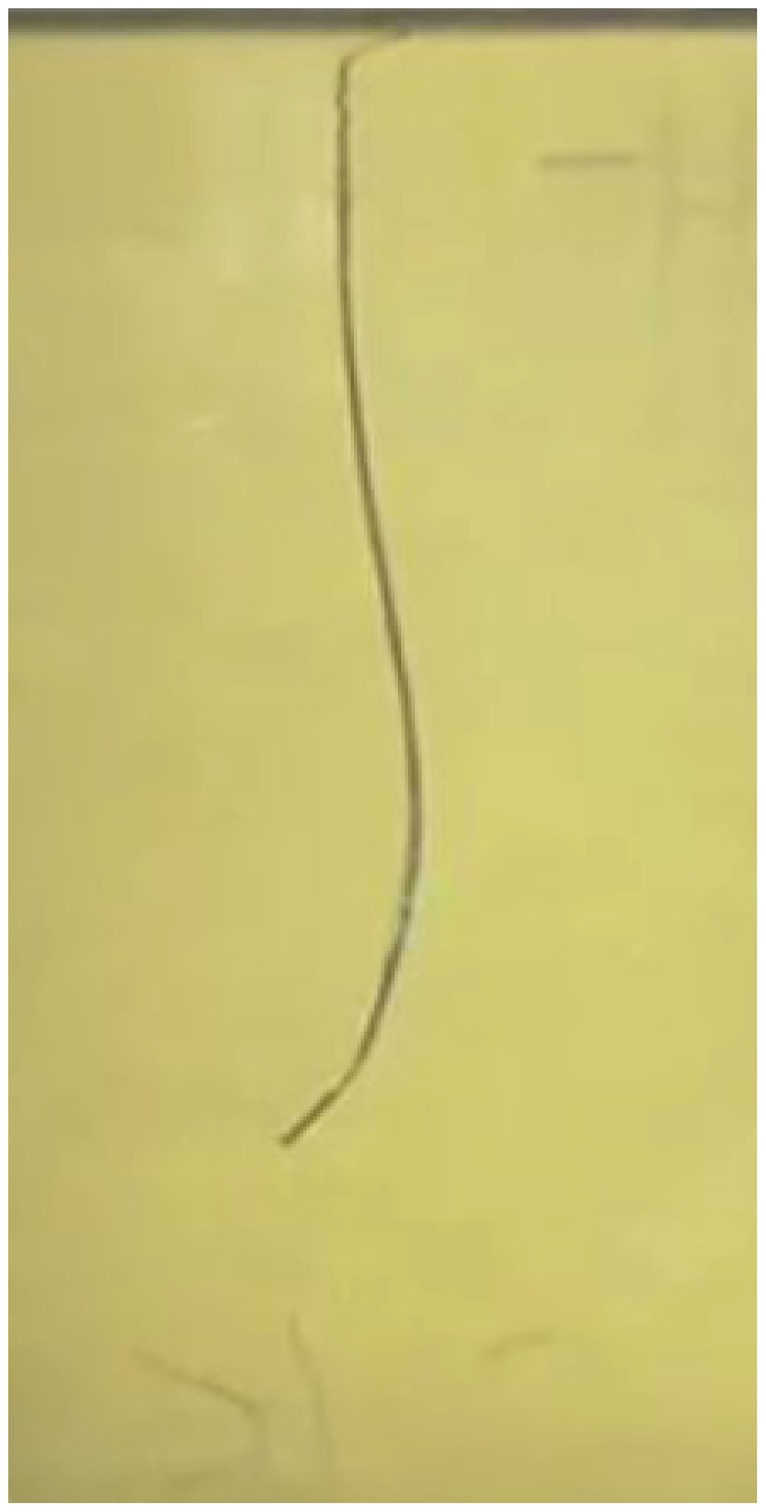
Probe following a multi-curve path.

During the experiments the probe was inserted in the gelatine using a constant wire retraction. For this reason, the probe followed only one curve in one direction. By changing the configuration of the wires during the insertion, the probe was able to follow a multi-curved path as shown in Fig 13 (S1 Video).

### Limitations and future work

In order to prevent buckling during the insertion of the probe in the gelatine phantom, two concentric tubes were used to support the shaft of the probe that was outside the gelatine. To avoid friction between the probe and these tubes, we chose 0.6 mm as inner diameter of the smallest tube. This left room for some small lateral displacement of the probe, which might have led to inaccuracy in the needle insertion.

We investigated the performance of the probe in homogeneous 10% wt gelatine phantoms (17 kPa, which might correspond to skeletal muscle tissue [37]). However, biological tissue is rather non-homogeneous, with common stiffness degrees ranging between 1 kPa (corresponding to brain tissue [33, 34]) and 50 kPa (corresponding to tendons [33, 34]). Multi-layered phantoms using different gelatine concentrations might mimic better the human anatomy. Additionally, after further development of the prototype, ex-vivo and in-vivo experiments are needed to test the performance in a more realistic situation.

**Fig 14:**
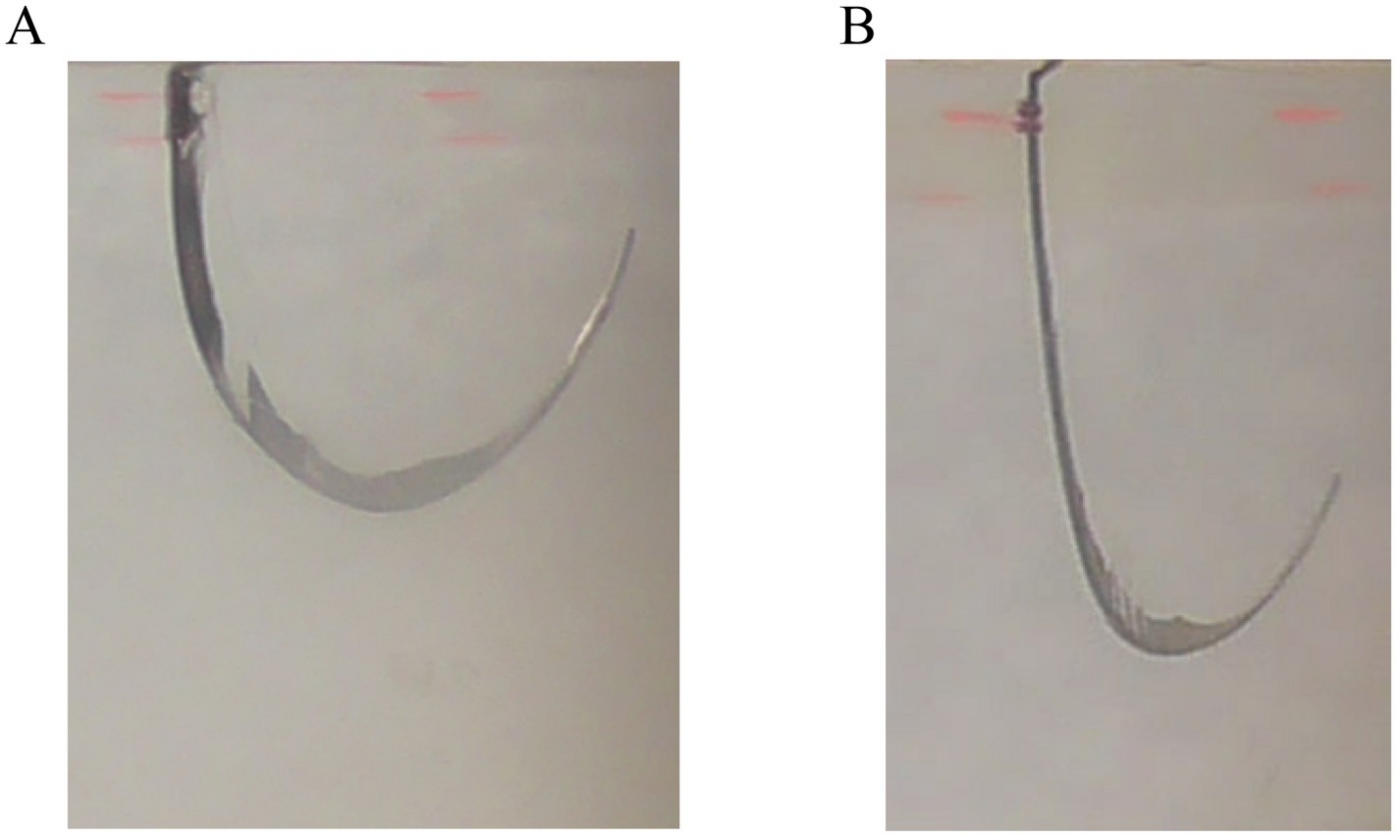
Probe cutting the gelatine during insertion (Experiment 2). (A) Condition 6 (W1 = L3, W2 = L3, and W3 = 0). (B) Condition 8 (W1 = L3, W2 = L1, and W3 = 0).

In some of the photographs we had difficulties in retrieving the probe because of low contrast or incorrect image focus. We performed a manual analysis of all the photographs, which limits the reproducibility of the study. When the probe was inserted into the gelatine with a high level of retraction (e.g. L3), we could notice that the probe was slicing through the substrate laterally along the path (Fig 14). This effect mostly occurred when the probe was following sharp curvatures and needs to be avoided during medical procedures [21].

The angle of the tip was set at the beginning of each test based on the turn of the wheel at the handle. However, during the insertion of the probe inside the gelatine, the tip angle might change because of the resistance forces acting from the gelatine on the tip. In future developments, a real-time measurement of the position of the probe tip could be used to investigate this aspect.

The tip of the probe consists of three wires bent and connected together. This design choice might not be ideal for a clinical setting. The empty space between the wires could entrap tissue during the insertion of the probe and in case of biopsy, healthy tissue could be contaminated. In future versions of the probe prototype, tissue trapping can be avoided by adding a cover around the tip (e.g., plastic sheath).

The tip of the prototype did not show any plastic deformation during all the measurements. It might be possible that because of the continuous retraction and bending, the wires will encounter plastic deformation over time. However, considering that the probe is meant for single use only, this possible effect is deemed not critical. Another risk for plastic deformation might occur when the probe encounters a stiff substrate (the yield load of the used Nitinol wires is 0.721 Kg as reported by the manufacturer). Future versions of the prototype would need to include a locking mechanism that protects the tip once a very stiff tissue is encountered.

In both experiments, a constant speed of 2 mm/s was used. In literature, there is not much information about the needle insertion speed used in clinical practice. Van Gerwen et al. [45] reported recorded speeds between 0.4 mm/s and 10 mm/s for epidural procedures [46] and much higher speeds of about 500 mm/s measured during brachytherapy interventions [47]. It is known that the friction force between the substrate and the needle increases with the insertion speed. Kaboyashi et al. [48] showed that when inserting a 17 G needle into porcine liver at different speeds, the friction force increased with speeds up to 2 mm/s, reaching a constant value for higher speeds. Mahvash et al. [49] observed that high needle insertion speeds are related to low tissue deformation and damage. However, safely insertion of a needle manually at high speeds might entail a risk of imprecise movement.

In this work, we assessed the performance of the probe by looking at the final position reached at the end of each measurement. In future work a fine-grained analysis of the entire path followed by the prototype could be also conducted, to gain a better understanding of the behaviour of the probe inside the substrate.

In our prototype, steering is achieved by rotating a wheel. In future prototypes, an ergonomic handle can be added, with which the end user intuitively steers the probe, similar to the handle that Van de Berg et al. [25] used in their cable-actuated needle design. Additionally, there should be a rigid telescopic tube system connected to the handle to guide the probe when the probe is outside the substrate and to prevent buckling during the insertion phase.

### Possible applications of the steerable probe

Needles with diameters between 22 G (0.71 mm) and 27 G (0.41 mm) are used in a number of procedures, such as fine needle aspiration (FNA) [50] and regional or peripheral anaesthesia [51]. In FNA, 25-G or 22-G needles have exhibited higher diagnostic accuracy than the 19-G needles that are typically used in core biopsy [52]. Thin needles are generally less traumatic than thick needles, and complications, due to the accidental puncture of a vessel for example, are also reduced in the former case [50]. In spinal anaesthesia, needles ranging between 22 G (0.72 mm) and 27 G (0.41 mm) are commonly used [51]. It has been shown that the smaller puncture created by smaller spinal needles is associated with a decrease in incidences of postdural puncture headache caused by cerebral spinal fluid leakage [53]. Steinfeldt et al. [54] showed a correlation between larger needle diameter and increased nerve damage during nerve block procedures. In biopsy procedures, the size of the needle influences the size of the sample that can be retrieved. Small diameter (under 1 mm) needle are commonly used for fine needle aspiration cytology, where a very small sample for cytological exams is needed [55].

The probe design demonstrated in this paper can be combined with a guiding outer needle, as it is currently done in the coaxial technique for FNA [56]. In this case, a standard 18–19 G rigid needle can be used to penetrate the first layers of tissues, after which the proposed probe, surrounded by a flexible tube, could be fed through the needle, pushed out of the tube and steered towards the target. In order to continuously track the position of the probe, CT imaging or ultrasound could be used, as with standard needles. Once the needle reaches the target, the flexible tube could be advanced and the probe could be withdrawn. The empty tube left in place could be used to extract tissue sample and inject drugs.

Next to a small diameter, needle steerability is an important property for improving accuracy and precision in reaching the desired target inside the body. The ability of changing the needle tip direction on-demand once the needle has been inserted into the tissue, without the need of axial rotation, could be beneficial for controllability. Steering can also be used to correct for unwanted needle deflection upon insertion into the tissue, or to adjust the trajectory if the target is moving due to tissue motion (e.g. breathing or heart biting).

Having an ultra-thin probe (diameter below 1 mm) that is able to steer, such as the probe presented in this work, can open new possibilities for the physician during complex procedures. One example is the celiac plexus blockage or neurolysis [57]. This is a procedure where medication is injected to relieve abdominal pain caused, for example, by pancreas cancer. The needle is inserted either from the back or the abdomen of the patient to reach the area behind the vertebra, avoiding to puncture organs and major vessels [58]. In the future, a needle as the one presented in our work could be used in combination with a thin-walled cannula, to access the fat space around the celiac plexus block, while avoiding to puncture organs or major blood vessels.

## Conclusion

This paper presents the design and evaluation of a 0.5-mm steering probe. The novel prototype combines the simple design of pre-curved needles with the steering method of cable-actuated needles. We showed that the probe was able to penetrate a gelatine phantom (10% wt) and steer omnidirectionally without the need of axial rotation. The probe was able to steer in the direction of the retracted wire, and the final position reached varied between small deflections from the straight path when the wires were slightly retracted and sharp curvatures when the wires were retracted more. The probe could be used in percutaneous procedures, such as spinal anaesthesia and fine needle aspiration, where a diameter smaller than 1 mm is desired. The ability to steer allows for correcting for small deviations from the path and for avoiding obstacles while navigating towards a target.

## Supporting information

S1 Appendix Probe tip selection. (DOCX)

S1 Video Video of the probe following a multi-curved path. (MP4)
