# Supplementary material for: Design of an ultra-thin steerable probe for percutaneous interventions and preliminary evaluation in a gelatine phantom
Source: PLoS One. 2019 Sep 4;14(9):e0221165. doi: 10.1371/journal.pone.0221165 (PMC6726204; doi:10.1371/journal.pone.0221165)
Supplement: S1 Appendix — (DOCX) [file pone.0221165.s001.docx]

# S1 Appendix. Probe tip selection

The choice for the tip design was made based on the results of a test where four prototypes were compared in terms of the pulling force required for bending at a maximum angle of 90°. Our goal was to identify which design allows for tip bending using the least amount of force.

## Test prototypes

Off-the-shelf Nitinol wires were used as elements (Flexmet, Belgium). Two Nitinol wires (0.125 mm diameter) were attached to each other at their distal end by gluing a stainless steel tube (ID=0.3 mm, OD=0.5 mm) over the wires. Directly after the application of the glue, the stainless steel tube was compressed with nose pliers to clamp the wires inside the tube. The curve close to the tip was achieved in three different ways: one curve, two-curves, and three-curves (Fig 1). A control design where the wires are not curved at the tip was also prepared. The three different prototypes contain pre-stresses which might influence the force of actuation needed to bend the tip as well as the final bending angle.

A
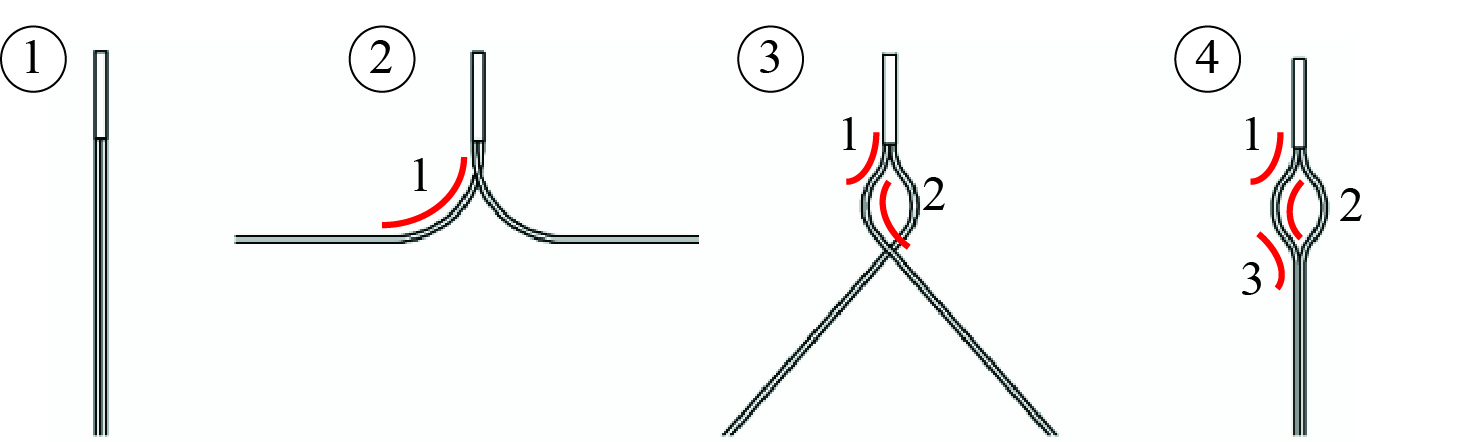


B
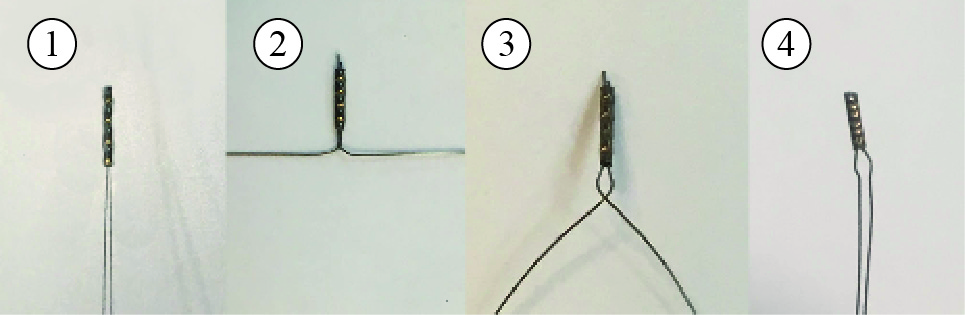


**Fig 1. Tip prototypes.**

(A) Drawing of the four prototypes showing the number of curves. 1) no curve, 2) one-curve, 3) two-curves, and 4) three-curves. (B) Photographs of the corresponding prototypes with two Nitinol wires connected at the tip with a stainless steel tube.

## Experimental setup & procedure

A linear stage (Thorlabs MTS25-Z8, 25 mm) with a load cell (FUTEK model LSB200 FSH00102 2 lb) was used to measure the retraction force needed to bend the tip of each prototype (Fig 2). One wire was fixed to a 3D printed support while the other one was connected to the linear stage. A video camera (Panasonic HC-V250) was positioned in front of the linear stage. A total displacement of the linear stage of 1 mm was chosen to limit the bending of the tip to a maximum of 90°. The stage moved at a constant velocity of 0.1 mm/s. Each prototype was tested five times.

A
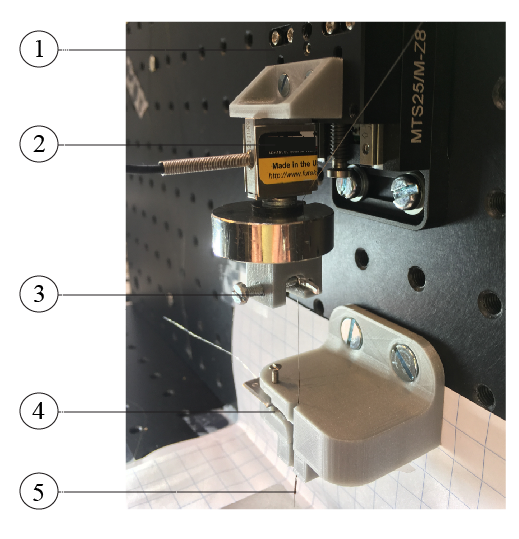
 B
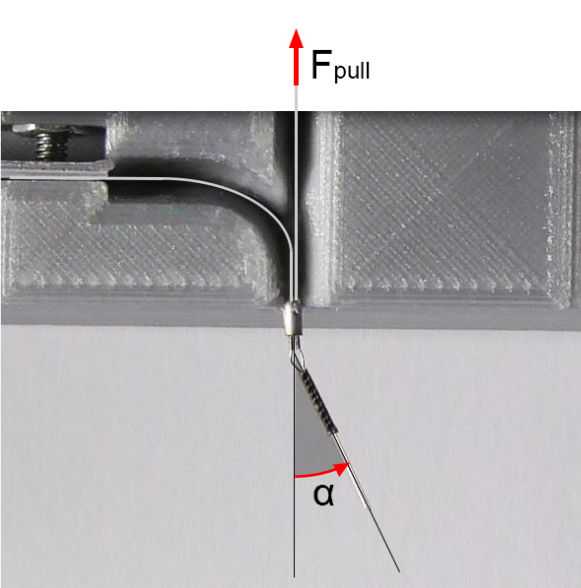


**Fig 2. Experimental setup.**

(A) close-up photo showing 1) motorized linear stage, 2) load cell Futek, 3) 3D printed connection for the wire connected to the load cell, 4) 3D printed connection for the wire connected to the linear stage, 5) tip of the prototype. (B) tip with two wires attached with at the 3D printed connection. One wire is pulled and the pulling force is measured ($F_{pull}$) while the other wire is clamped to the 3D printed part. The result of the wire being pulled is a bent of an angle α of the tip.

## Results

The prototype with no curve required the highest pulling force to bend (mean value = 2.57 N, standard deviation = 0.07 N, n =5), and the prototype with two-curves bent with the least pulling force (mean value = 0.85 N, standard deviation = 0.02 N, n = 5). One-curve and three-curves prototype showed similar results, 1.34 ± 0.02 N (mean ± standard deviation) and 1.32 ± 0.08 N (mean ± standard deviation), respectively. Based on these results, the design with two-curves at the tip was chosen for the tip of the prototype probe.
